# Supplementary material for: Population-based study of eclampsia: Lessons learnt to improve maternity care
Source: PLoS One. 2024 May 2;19(5):e0301976. doi: 10.1371/journal.pone.0301976 (PMC11065303; doi:10.1371/journal.pone.0301976)
Supplement: S1 Checklist — (DOCX) [file pone.0301976.s001.docx]

STROBE Statement—checklist of items that should be included in reports of observational studies

|  | Item No. | Recommendation | Page  No. | Relevant text from manuscript |
| --- | --- | --- | --- | --- |
| **Title and abstract** | 1 | (*a*) Indicate the study’s design with a commonly used term in the title or the abstract | 1 | Population-based study |
|  |  | (*b*) Provide in the abstract an informative and balanced summary of what was done and what was found | 2 | The high proportion of inadequate quality of care underlines the need for an evidence-based standardisation of care for HDP. |
| Introduction | | | |  |
| Background/rationale | 2 | Explain the scientific background and rationale for the investigation being reported | 3 | Previous studies have mainly focused on the management of the eclampsia event itself, in particular, the administration of magnesium sulphate in this context and showed there is room for improvement |
| Objectives | 3 | State specific objectives, including any prespecified hypotheses | 3-4 | The aims of this planned ancillary analysis were first to describe the characteristics of women with eclampsia and to compare them with those of women with non-eclamptic HDP-related SMM and of control women without SMM; and second, to analyse the adequacy of management in women who had eclampsia at different stages of their care pathway. These objectives were chosen to identify opportunities for primary and secondary prevention of eclampsia. |
| Methods | | | |  |
| Study design | 4 | Present key elements of study design early in the paper | 4 | The source population came from the EPIMOMS project, a prospective population-based study |
| Setting | 5 | Describe the setting, locations, and relevant dates, including periods of recruitment, exposure, follow-up, and data collection | 4 | It was conducted in six French regions from May 1, 2012, through November 30, 2013 (recruitment and data collection). Recruitment took place over a one-year period in each region at 119 maternity units and 136 intensive care units (regardless of the hospitals level of care, number of annual deliveries, or public or private status) that accounted for 182,309 maternities during the study period, i.e., one fifth of those in France during that period. |
| Participants | 6 | (*a*) *Cohort study*—Give the eligibility criteria, and the sources and methods of selection of participants. Describe methods of follow-up  *Case-control study*—Give the eligibility criteria, and the sources and methods of case ascertainment and control selection. Give the rationale for the choice of cases and controls  *Cross-sectional study*—Give the eligibility criteria, and the sources and methods of selection of participants | 5 | For this analysis, conducted in 2021, we selected 3 study groups: women with eclampsia (n=51), with each case of eclampsia reviewed to ensure the accuracy of the diagnosis; women with SMM related to a non-eclamptic HDP (n=351), and the women without SMM in the EPIMOMS control group (n=3,651). |
|  |  | (*b*) *Cohort study*—For matched studies, give matching criteria and number of exposed and unexposed  *Case-control study*—For matched studies, give matching criteria and the number of controls per case |  |  |
| Variables | 7 | Clearly define all outcomes, exposures, predictors, potential confounders, and effect modifiers. Give diagnostic criteria, if applicable | 4 | Eclampsia was defined by the occurrence of seizures in a woman diagnosed with pre-eclampsia, or whose seizures could not be attributed to another cause, in accordance with the national guidelines on multidisciplinary management of severe pre-eclampsia |
| Data sources/ measurement | 8* | For each variable of interest, give sources of data and details of methods of assessment (measurement). Describe comparability of assessment methods if there is more than one group | *5* | *These data were collected by a manual review of medical records by research midwives trained for this study and entered in a specific electronic report form developed for the study, and were anonym. Additionally, for women with SMM, the criteria, causes, clinical timing (antepartum, intrapartum or postpartum) and details about the course and management of the severe morbid event were prospectively collected by the clinician in charge.* |
| Bias | 9 | Describe any efforts to address potential sources of bias | NA |  |
| Study size | 10 | Explain how the study size was arrived at | NA |  |

Continued on next page

| Quantitative variables | 11 | Explain how quantitative variables were handled in the analyses. If applicable, describe which groupings were chosen and why | 8 | Proportions are presented as percentages and skewed distributions as medians with interquartile ranges (IQRs). Mean and Standard Deviation are presented for normal distributions. |
| --- | --- | --- | --- | --- |
| Statistical methods | 12 | (*a*) Describe all statistical methods, including those used to control for confounding | 7-8 | The incidence of eclampsia was calculated as the number of women with eclampsia divided by the total number of women who gave birth in the participating maternity units during the inclusion period, with a binomial 95% confidence interval.  The socio-demographic characteristics of the women, their medical and obstetric histories, and the characteristics of their pregnancy were described for each group and compared: the women with eclampsia, those with SMM related to non-eclamptic HDP and the representative sample of women without SMM.  For women with eclampsia, we described the timing of eclampsia occurrence, the mode of and gestational age at birth, the clinical and laboratory characteristics during the period preceding eclampsia and during the eclampsia episode, the management of eclampsia, and the maternal and neonatal complications.  The quality of care in women with eclampsia, in the 5 categories described above, was reported for each of the three stages of management.  Proportions are presented as percentages and skewed distributions as medians with interquartile ranges (IQRs). Mean and Standard Deviation are presented for normal distributions. The comparisons of characteristics between groups were tested with Student tests, Chi2, Fisher exact tests, as appropriate. All analyses were carried out with STATA v13 software (StataCorp, College Station, TX, USA). |
|  |  | (*b*) Describe any methods used to examine subgroups and interactions | NA |  |
|  |  | (*c*) Explain how missing data were addressed | NA |  |
|  |  | (*d*) *Cohort study*—If applicable, explain how loss to follow-up was addressed  *Case-control study*—If applicable, explain how matching of cases and controls was addressed  *Cross-sectional study*—If applicable, describe analytical methods taking account of sampling strategy | NA |  |
|  |  | (*e*) Describe any sensitivity analyses | NA |  |
| Results | | | | |
| Participants | 13* | (a) Report numbers of individuals at each stage of study—eg numbers potentially eligible, examined for eligibility, confirmed eligible, included in the study, completing follow-up, and analysed | 5 | For this analysis, conducted in 2021, we selected 3 study groups: women with eclampsia (n=51), with each case of eclampsia reviewed to ensure the accuracy of the diagnosis; women with SMM related to a non-eclamptic HDP (n=351), and the women without SMM in the EPIMOMS control group (n=3,651). |
|  |  | (b) Give reasons for non-participation at each stage | NA |  |
|  |  | (c) Consider use of a flow diagram | NA |  |
| Descriptive data | 14* | (a) Give characteristics of study participants (eg demographic, clinical, social) and information on exposures and potential confounders | 8-9 | Women with eclampsia were more often born outside Europe, compared with both the women with SMM related to non-eclamptic HDP and women without SMM (Table 1); the proportion of women born in sub-Saharan Africa was particularly high in the eclampsia group (20.0% compared with 15.0% and 5.7% respectively). Women with eclampsia also more often had no source of legal work-related income and no standard health insurance and more often lived without a partner, although the difference with women with non-eclamptic HDP-related SMM did not reach statistically significance.  In addition, compared with women without SMM, women with eclampsia and those with non-eclamptic HDP-related SMM had a higher prevalence of classical risk factors for HDP: chronic hypertension, gestational hypertensive disorder in a previous pregnancy, and multiple pregnancies. |
|  |  | (b) Indicate number of participants with missing data for each variable of interest | NA |  |
|  |  | (c) *Cohort study*—Summarise follow-up time (eg, average and total amount) | NA |  |
| Outcome data | 15* | *Cohort study*—Report numbers of outcome events or summary measures over time | *8* | *Fifty-one cases of eclampsia occurred, for a population-based incidence of eclampsia of 2.8 per 10,000 (95% CI 2.0–4.0).* |
|  |  | *Case-control study—*Report numbers in each exposure category, or summary measures of exposure | *NA* |  |
|  |  | *Cross-sectional study—*Report numbers of outcome events or summary measures | *NA* |  |
| Main results | 16 | (*a*) Give unadjusted estimates and, if applicable, confounder-adjusted estimates and their precision (eg, 95% confidence interval). Make clear which confounders were adjusted for and why they were included | NA |  |
|  |  | (*b*) Report category boundaries when continuous variables were categorized | NA |  |
|  |  | (*c*) If relevant, consider translating estimates of relative risk into absolute risk for a meaningful time period | NA |  |

Continued on next page

| Other analyses | 17 | Report other analyses done—eg analyses of subgroups and interactions, and sensitivity analyses | NA |  |
| --- | --- | --- | --- | --- |
| Discussion | | | | |
| Key results | 18 | Summarise key results with reference to study objectives | 18 | In this population-based study 1 out of every 3,600 women who gave birth had eclampsia. Socially vulnerable subgroups were more represented among women with eclampsia than among those with non-eclamptic HDP-related SMM or control women, in particular migrant women from sub-Saharan Africa. The care provided to women with eclampsia was completely inadequate or substandard for the majority of them and suggests major opportunities for improvement at two main stages: in the management of pre-eclampsia before eclampsia and in the management of the eclamptic episode. In particular, we found an inappropriately low rate of magnesium sulphate use for both primary and secondary prevention of eclampsia. |
| Limitations | 19 | Discuss limitations of the study, taking into account sources of potential bias or imprecision. Discuss both direction and magnitude of any potential bias | 21 | Because of the rarity of the event, the number of eclampsia cases limited the analysis of quality of care for some subgroups of women with reported high-risk profiles, notably women born in sub-Saharan Africa. The EPIMOMS study was conducted 8 years ago, and practices may have changed. However, although the national guidelines for pre-eclampsia management were updated in 2021, we believe the analysis of the quality of care assessed from the EPIMOMS data, and the opportunities for improvement identified, are still relevant. Indeed, the recent guidelines are similar to the previous ones for the components of care assessed in our analysis, except for the prophylactic use of magnesium sulphate, which is now recommended for even broader clinical symptoms not restricted to neurological signs, as in the most recent international guidelines. |
| Interpretation | 20 | Give a cautious overall interpretation of results considering objectives, limitations, multiplicity of analyses, results from similar studies, and other relevant evidence | 21 | This population-based study of women with eclampsia underlines the need to improve the quality of care provided for HDP at all stages of the morbidity continuum and for all women. |
| Generalisability | 21 | Discuss the generalisability (external validity) of the study results | 21 | The EPIMOMS study was conducted 8 years ago, and practices may have changed. However, although the national guidelines for pre-eclampsia management were updated in 2021, we believe the analysis of the quality of care assessed from the EPIMOMS data, and the opportunities for improvement identified, are still relevant. Indeed, the recent guidelines are similar to the previous ones for the components of care assessed in our analysis, except for the prophylactic use of magnesium sulphate, which is now recommended for even broader clinical symptoms not restricted to neurological signs, as in the most recent international guidelines. |
| Other information | |  | | |
| Funding | 22 | Give the source of funding and the role of the funders for the present study and, if applicable, for the original study on which the present article is based |  | Supported by a grant from the National Research Agency and the Île-deFrance Regional Health Agency. |

*Give information separately for cases and controls in case-control studies and, if applicable, for exposed and unexposed groups in cohort and cross-sectional studies.

**Note:** An Explanation and Elaboration article discusses each checklist item and gives methodological background and published examples of transparent reporting. The STROBE checklist is best used in conjunction with this article (freely available on the Web sites of PLoS Medicine at http://www.plosmedicine.org/, Annals of Internal Medicine at http://www.annals.org/, and Epidemiology at http://www.epidem.com/). Information on the STROBE Initiative is available at www.strobe-statement.org.
