# Supplementary material for: Population-based study of eclampsia: Lessons learnt to improve maternity care
Source: PLoS One. 2024 May 2;19(5):e0301976. doi: 10.1371/journal.pone.0301976 (PMC11065303; doi:10.1371/journal.pone.0301976)
Supplement: S1 Fig — (DOCX) [file pone.0301976.s004.docx]

Figure S1: Gestational age at the time of the eclamptic episode (for women with antepartum or intrapartum seizures) or at delivery (for women with postpartum seizures)
